# Molecular survey of *Bartonella* spp., *Ehrlichia* spp., *Anaplasma* spp. and hemoplasmas in small synanthropic mammals in urban areas of Brazil’s northern Amazon region

**DOI:** 10.1590/S1984-29612025056

**Published:** 2025-10-20

**Authors:** Darlison Chagas-de-Souza, Cláudia Regina Silva, Tássio Alves-Coêlho, Anna Claudia Baumel Mongruel, Clara Morato Dias, Paulo Vitor Cadina Arantes, Marcos Rogério André, Lúcio André Viana

**Affiliations:** 1 Universidade Federal do Amapá – UNIFAP, Programa de Pós-Graduação em Biodiversidade Tropical, Macapá, AP, Brasil; 2 Universidade Federal do Amapá – UNIFAP, Departamento de Ciências Biológicas e da Saúde, Laboratório de Estudos Morfofisiológicos e Parasitários, Macapá, AP, Brasil; 3 Instituto de Pesquisas Científicas e Tecnológicas do Estado do Amapá – IEPA, Laboratório de Mastozoologia, Macapá, AP, Brasil; 4 Universidade Estadual “Júlio de Mesquita Filho” – UNESP, Faculdade de Ciências Agrárias e Veterinárias – FCAV, Departamento de Patologia, Reprodução e Saúde Única, Laboratório de Bioagentes Transmitidos por Vetores, Jaboticabal, SP, Brasil

**Keywords:** Mycoplasma, Anaplasmataceae, neglected diseases, Brazilian Amazon, forests fragments, Mycoplasma, Anaplasmataceae, doenças negligenciadas, Amazônia brasileira, fragmentos florestais

## Abstract

Representatives of the families Didelphidae, Echimyidae, Cricetidae, and Muridae have been identified as significant reservoirs or amplifiers of zoonotic agents. This study aimed to investigate the molecular occurrence of *Bartonella* spp., *Ehrlichia* spp., *Anaplasma* spp., and hemotropic *Mycoplasma* spp. (hemoplasmas) in small synanthropic mammals in different urban complexes in Brazil’s northern Amazon region. Between January and August 2022, blood samples were collected from 36 small mammal specimens, belonging to ten different species living in three vegetation fragments located in the metropolitan areas of Macapá, in the state of Amapá, and in Santarém and Marabá, state of Pará, Brazil. After DNA extraction, samples were subjected to real-time quantitative PCR (qPCR) for *Bartonella* spp. based on the *nuoG* gene, and to conventional PCR assays for *Ehrlichia* spp., *Anaplasma* spp., and hemoplasmas based on the *dsb* and 16S rRNA genes, respectively. This is the first record of *Anaplasma* spp. and hemotropic *Mycoplasma* sp. in marsupials in northern Brazil. *Bartonella* spp. was detected only in small mammals from Macapá, expanding the list of known hosts. This study describes findings on potentially zoonotic pathogens associated with small mammals living in green areas of large urban complexes in the Brazilian Amazon.

## Resumo

Representantes das famílias Didelphidae, Echimyidae, Cricetidae e Muridae foram identificados como reservatórios ou amplificadores significativos de agentes zoonóticos. Este estudo teve como objetivo investigar a ocorrência molecular de *Bartonella* spp., *Ehrlichia* spp., *Anaplasma* spp. e *Mycoplasma* spp. hemotrópicos (hemoplasmas) em pequenos mamíferos sinantrópicos em diferentes complexos urbanos no norte da Amazônia brasileira. Entre janeiro e agosto de 2022, amostras de sangue foram coletadas de 36 espécimes de pequenos mamíferos, pertencentes a dez espécies, habitando três fragmentos de vegetação localizados nas regiões metropolitanas de Macapá, no estado do Amapá, e de Santarém e Marabá, no estado do Pará, Brasil. Após extração de DNA, as amostras foram submetidas à PCR quantitativa em tempo real para *Bartonella* spp. com base no gene *nuoG*, e à PCR convencional para *Ehrlichia* spp., *Anaplasma* spp. e hemoplasmas, utilizando os genes *dsb* e 16S rRNA,. Este é o primeiro registro de *Anaplasma* spp. e *Mycoplasma* sp. hemotrópico em marsupiais no norte do Brasil. *Bartonella* spp. foi detectada apenas em pequenos mamíferos de Macapá, ampliando a lista de hospedeiros conhecidos. Este estudo descreve achados de patógenos potencialmente zoonóticos associados a pequenos mamíferos que habitam áreas verdes de grandes complexos urbanos na Amazônia brasileira.

## Introduction

Research into neglected, emerging, and re-emerging diseases gained renewed attention in the early 21^st^ century. Research in this field gained further relevance due to by the COVID-19 pandemic in March 2020 (WHO, 2020). A six-decade study (1940-2000) identified 335 emerging infectious disease events, 78.1% from wildlife, of which 54.3% were caused by bacterial or rickettsial agents (Jones et al., 2008).

According to Taylor et al. (2001), approximately 75% of emerging infectious diseases are represented by zoonoses, most of which are vectored by arthropods. The detection of these zoonotic agents is linked to the use of new diagnostic tools, particularly the development of molecular methods, but their emergence is primarily due to human behavior and changes in natural habitats. Moreover, close contact with wild animals increases the likelihood of zoonotic disease emergence (Chomel et al., 2007).

A variety of free-living animals, such as rodents, bats, shrews, rabbits and didelphids, are known to be involved in the transmission cycles of potentially pathogenic agents of the family Bartonellaceae (Breitschwerdt, 2014). Didelphids have been identified as potential amplifiers of some pathogens, such as those of the family Rickettsiaceae (Horta et al., 2010), as well as putative reservoirs of *Ehrlichia* spp. and hemotropic *Mycoplasma* (Pontarolo et al., 2021; André et al., 2022; Braga et al., 2023; Oliveira et al., 2023b).

*Bartonella* spp. (Hyphomicrobiales: Bartonellaceae) comprise fastidious, Gram-negative, and facultative intracellular bacteria that parasitize mainly erythrocytes and endothelial cells. They can also colonize dendritic cells and macrophages of many mammals, and some bacterial species are zoonotic (Eicher & Dehio, 2012). These agents, whose main reservoirs are mammals, can be transmitted by various hematophagous arthropods, depending on the species involved, such as sandflies (*Lutzomyia verrucarum*), lice (*Pediculus humanus corporis*), fleas (*Ctenocephalides felis*, *Ctenophthalmus nobilis, Pulex irritans*), and possibly ticks (*Ixodes ricinus* and *Rhipicephalus sanguineus*) (Chomel et al., 2009; Breitschwerdt et al., 2010; Tsai et al., 2011; Silaghi et al., 2016; Breitschwerdt, 2017; Wechtaisong et al., 2020).

*Anaplasma* spp. and *Ehrlichia* spp. (Rickettsiales: Anaplasmataceae) are tick-borne obligate intracellular bacteria of notable importance in veterinary and human medicine, with cosmopolitan distribution and potentially zoonotic agents (André, 2018). They are small Gram-negative, pleomorphic bacteria with morphology varying from coccoid to ellipsoidal, parasites of blood cells (Dumler et al., 2001; Dumler, 2005). In natural environments, these microorganisms remain in ticks, with small and large terrestrial mammals as reservoirs (Kawahara et al., 2006).

Hemotropic mycoplasmas (hemoplasmas) (Mycoplasmatales: Mycoplasmataceae) are Gram-negative pleomorphic bacteria lacking cell walls, which adhere to the erythrocyte surface of mammals, causing hemolytic anemia in varying degrees (Messick, 2004). The clinical spectrum of infection ranges from asymptomatic to life-threatening, depending partly on host susceptibility. Reported non-specific clinical signs include lethargy, anorexia, fever and anemia (Messick, 2004; Millán et al., 2021). Hemoplasmas are capable of infecting domestic, wild and livestock animals (Millán et al., 2021). Human infections have been reported mainly in immunocompromised patients (Steer et al., 2011; Descloux et al., 2021; Hattori et al., 2020; Alcorn et al., 2021). The transmission routes of hemoplasmas have yet to be fully identified (Millán et al., 2024). However, hemoplasma DNA has been found in hematophagous arthropods such as ticks, flies, and fleas (Cevidanes et al., 2023; Descloux et al., 2021; Gonçalves et al., 2020). The vertical transmission of these bacteria has been suggested in humans, domestic animals and wild animals, despite limited evidence (Steer et al., 2011; Sasaoka et al., 2015; Andrade et al., 2024; Millán et al., 2024). To date, congenital transmission has not been proven even through experimental infection (Cohen et al., 2018).

Infections caused by these organisms in reservoir hosts are characterized by low levels of bacteria in the bloodstream, which makes it difficult to detect them using traditional methods such as light microscopy. Therefore, these infections are usually diagnosed using polymerase chain reaction (PCR). Despite the worldwide occurrence of such agents in wild animals, only a few studies in Brazil have reported the occurrence and genetic diversity of *Bartonella* spp. (Gonçalves et al., 2016, 2020; Sousa et al., 2018; Amaral et al., 2022a, b; Braga et al., 2023; Pacheco et al., 2024), *Anaplasma* spp., *Ehrlichia* spp. (Sousa et al., 2017; Benevenute et al., 2017; André et al., 2022; Braga et al., 2023), and hemoplasmas (Gonçalves et al., 2015; 2020; Pontarolo et al., 2021; Braga et al., 2023; Oliveira et al., 2023a; Machado et al., 2024) in small non-volant mammals in this country. The present study aimed to investigated the molecular occurrence of such agents in small synanthropic mammals in different urban complexes in Brazil’s northern Amazon region.

## Material and Methods

### Study area and sampling of small non-volant mammals

**Figure 1:**
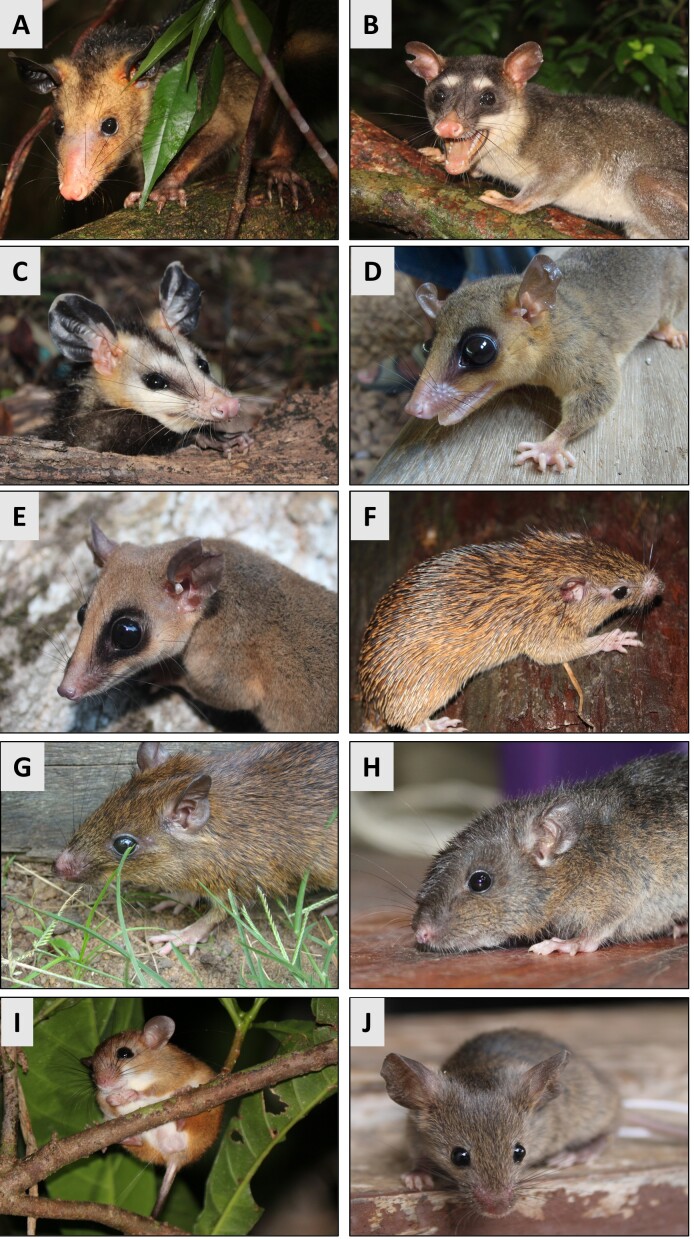
Small mammals sampled in the eastern Amazon for a molecular survey of *Bartonella* spp., hemotropic *Mycoplasma* spp., *Anaplasma* spp., and *Ehrlichia* spp. (A) *Didelphis marsupialis*. (B) *Philander opossum*. (C) *Didelphis imperfecta*. (D) *Marmosa demerarae*. (E) *Marmosa murina*. (F) *Mesomys hispidus*. (G) *Proechimys guyannensis*. (H) *Nectomys rattus*. (I) *Oecomys bicolor*. (J) *Mus musculus.*

**Figure 2:**
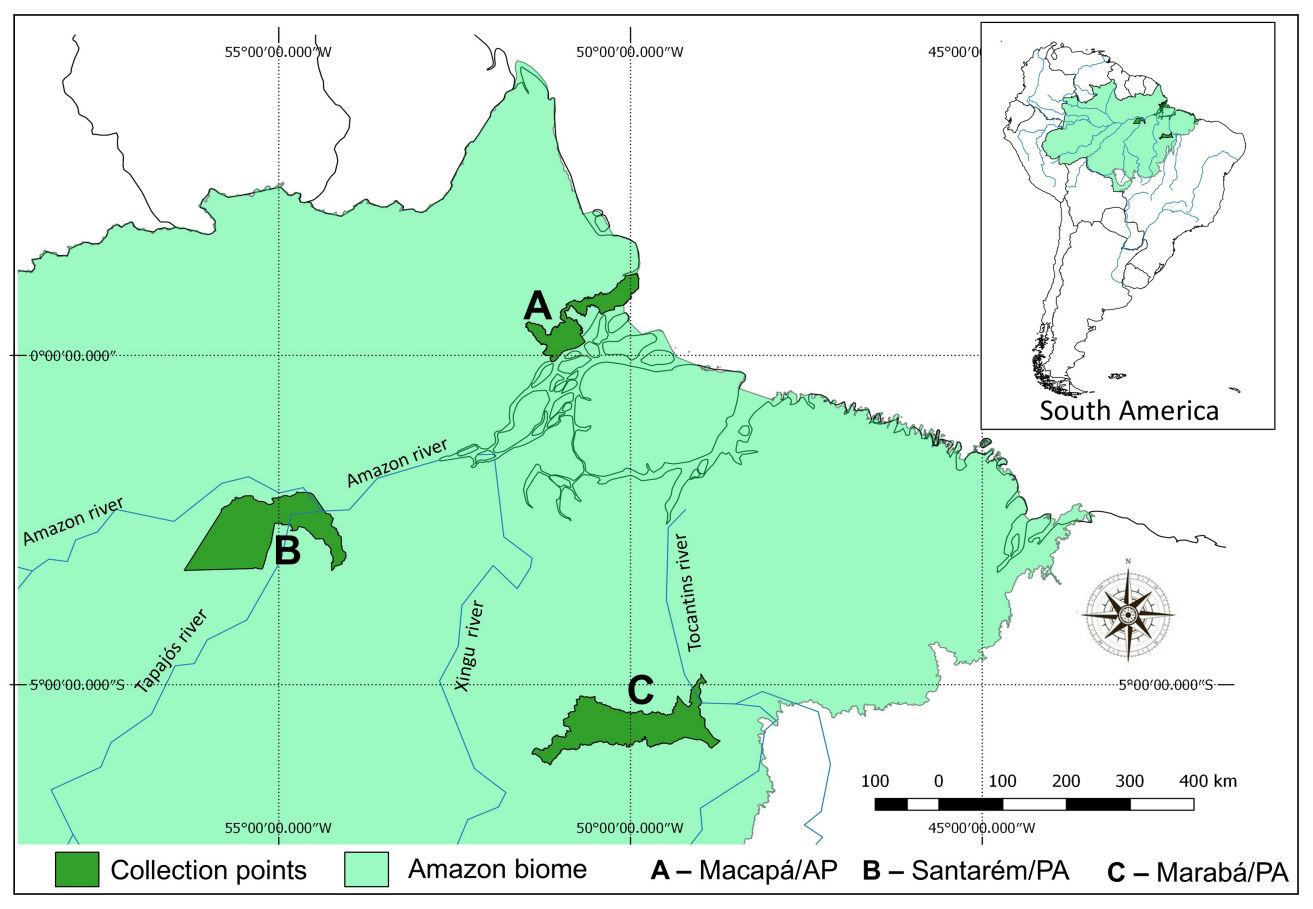
Map of the location of the sampling points in Brazil’s eastern Amazon region.

Between January and August 2022, 36 specimens of ten species of small mammal were captured in three vegetation fragments in urban areas of the eastern Amazon region (Figure 1). The sampling sites are located in the metropolitan area of Macapá, state of Amapá – AP (0°1’7.82”S, 51° 7’34.19”W), the metropolitan area of Santarém, state of Pará – PA (2°25’3.99”S 54°44’19.47”W), and in the industrial area of the municipality of Marabá, PA (5°25’21.87”S, 49°7’10.83”W) (Figure 2). The small mammals were captured using pitfall traps and live traps, specifically Tomahawk and Sherman traps.

In accordance with capture authorizations, female specimens with offspring were not handled for biological sample collection, and recaptures were disregarded, thus the totality of captured animals was not used for molecular detection of hemotropic bacteria. The captured animals were physically restrained to draw blood samples through tail venipuncture.

A portion of each blood sample was used to prepare duplicate blood smears, while another was preserved in a solution containing 96% alcohol. The samples were sent to the Vector-Borne Bioagents Laboratory at the Faculty of Agricultural and Veterinary Sciences (FCAV) of Universidade Estadual Paulista (UNESP) 'Júlio de Mesquita Filho' in Jaboticabal (state of São Paulo) for molecular detection and characterization procedures.

Blood smears were stained with Quick Panoptic stain/LABORCLIN® and examined under light microscopy under 400x and 1000x magnification at the Laboratório de Microscopia e de Coleta de Amostras of the Universidade Federal do Oeste do Pará – UFOPA.

In addition, two out of every 10 collected specimens were anesthetized and then euthanized with an overdose of 10% ketamine hydrochloride (100 mg/ml) and 1% acepromazine (10 mg/ml). These specimens were deposited as voucher specimens in the Coleção de Mamíferos in the Coleção Científica da Fauna do Amapá (CCFA).

### DNA extraction and amplification of mammalian endogenous gene

DNA was extracted from blood samples using a commercial extraction kit (DNeasy Blood & Tissue, Qiagen, Hilden, Germany), following the manufacturer’s instructions. The successful extraction of genetic material was ensured by subjecting the samples to endogenous gene amplification to check for the presence of amplifiable DNA from the mammalian host. To this end, DNA samples were subjected to a conventional PCR (polymerase chain reaction) protocol based on a 450 base pair (bp) fragment of the endogenous mammalian glyceraldehyde-3-phosphate dehydrogenase (*gapdh*) gene. The primers used here were GAPDH-F (5’-CCTTCATTGACCTCAACTACAT-3’) and GAPDH-R (5’- CCAAAGTTGTCATGGATGACC-3’), following the protocol described by Birkenheuer et al. (2003). The amplification reaction was carried out using a final volume of 25 μL, containing a mixture of 5 μL of DNA sample, 0.2 mM of each deoxynucleotide (dNTP), 0.4 μM of each primer oligonucleotide, 3, 0 mM Magnesium Chloride, 1.25 U Platinum^TM^ Taq DNA Polymerase (Life Technologies®, Carlsbad, California, United States), 10X buffer was used at a final concentration of 1× in the PCR reaction (PCR Buffer 10X- 100nM Tris-HCl, pH 9.0, 500 mM KCl) and ultrapure sterile water q.s.p. The temperature protocol and cycles used in the thermal cycler for each reaction were as follows: initial denaturation at 95°C for 5 minutes, 35 cycles consisting of denaturation at 95°C for 15 seconds, annealing at 50°C for 30 seconds, extension at 72°C for 30 seconds, and final extension at 72°C for 5 minutes. Positive samples in the *gapdh*-based PCR protocol were subjected to molecular assays for hemoplasmas, *Ehrlichia* spp., *Anaplasma* spp., and *Bartonella* spp.

### Molecular assays for hemotropic *Mycoplasma* spp.

A semi-nested PCR based on the 16S rRNA gene (1,107 bp) was used as the hemoplasma molecular detection assay (Harasawa et al., 2014; Di Cataldo et al., 2020). Positive samples were subjected to PCR targeting 800 bp of the 23S rRNA gene of hemoplasmas (Mongruel et al., 2022) for additional molecular characterization. *Mycoplasma ovis* DNA obtained from a naturally infected sheep was used as a positive control (Mongruel et al., 2022). Ultrapure sterile water (Promega, Madison, Wisconsin, USA) free of DNAses and RNAses was used as negative control in all the reactions. PCR primers and conditions are described in Table 1.

**Table 1:** Technical conditions of PCR reactions for hemotropic *Mycoplasma* spp., *Bartonella* spp., *Ehrlichia* spp., and *Anaplasma* spp.

**Gene**	**Oligonucleotide sequences**	**Thermocycling conditions s**	**References**
			
Hemotropic *Mycoplasma* spp.			
			
	1^st^ round:	95°C for 5 min, followed by 35 cycles of denaturation at 95°C for 30 sec, annealing at 57ºC for 30 sec, extension at 72°C for 1 min, and final extension at 72°C for 10 min for both rounds.	Harasawa et al. (2014), Di Cataldo et al. (2020)
	5'-AGAGTTTGATCCTGGCTCAG -3'		
**16S rRNA**	5'- TACCTTGTTACGACTTAACT-3'		
	2^nd^ round:		
	5'-ATATTCCTACGGGAAGCAGC -3'		
	5'-TACCTTGTTACGACTTAACT -3		
		94°C for 3 min, followed by cycles of denaturation at 94°C for 30 sec, annealing at 54oC for 30 sec, extension at 72°C for 1 min, and final extension at 72°C for 10 min.	
	5'-TGAGGGAAAGAGCCCAGAC-3'		
**23S rRNA**	5'GGACAGAATTTACCTGACAAGG-3'		Mongruel et al. (2022)
		
		
		
*Ehrlichia* spp.			
		95°C for 2 min; followed by 50 cycles of 95°C for 30 sec, 55°C for 30 sec and 72°C for 1 min, and a final extension of 72°C for 5 min.	
**16S rRNA**	5'-GATGATGTCTGAAGATATGAAACAAAT-3'		
	5'- CTGCTCGTCTATTTACTTCTTAAAGT-3'		Correa et al. (2011)
	
	
	
			
			
*Anaplasma* spp.			
			
**16S rRNA**	5'-CACATGCAAGTCGAACGGATTC-3'	40 cycles for the first round and 30 cycles for the second with the following temperatures: 94°C for 5 min, 94°C for 30 sec and 55°C for 30 sec, 72°C for 1 min; and a final extension at 72°C for 5 min.	
	5'-TTCCGTTAAGAAGGATCTAATCTCC-3'		
	5'-AACGGATTATTCTTTATAGCTTGCT-3'		Massung et al. (1998)
	5'-GGCAGTATTAAAAGCAGCTCCAGG-3'		
			
			
*Bartonella* spp.			

***ribC***	5'-TAACCGATATTGGTTGTGTTGAAG-3'	95°C for 10 min; 37 cycles: 95°C for 1 min, 51°C for 1 min and 72°C for 1 min; 72°C for 3 min.	Johnson et al. (2003)
	5'-TAAAGCTAGAAAGTCTGGCAACATAACG-3'		
		
***gltA***	5'-GGGACCAGCTCATGGTGGC-3'	95°C for 10 min; 35 cycles: 95°C for 20 sec, 51°C for 30 sec and 72°C for 2 min; 72°C for 3 min.	Norman et al. (1995)
	5'- AATGCAAAAGAACAGTAAACA-3'		
		
			
***rpoB***	5'-GCACGATTYGCATCATCATTTTCC-3'5'-CGCATT ATGGTCGTATTTGTCC-3'	94°C for 2 min; 40 cycles: 94°C for 45 sec, 52°C for 45 sec and 72°C for 45 sec; 72°C for 7 min.	Paziewska, et al. (2011)
***groEL***	5'-GGAAAAAGTGGGCAATGAAG-3'	94°C for 2 min; 40 cycles: 94°C for 45 sec, 47°C for 45 sec and 72°C for 45 sec; 72°C for 7 min.	Paziewska, et al. (2011),
	5'-TCCTTTAACGGTCAACGCATT-3'		Zeaiter et al. (2002)
		
			
**ITS 16S - 23S rRNA**	5'-CTTCAGATGATGATCCCAAGCCTTTTGGCG-3'	95°C for 2 min; 55 cycles: 94°C for 15 sec, 66°C for 15 sec and 72°C for 18 sec; 72°C for 1 min.	Maggi et al. (2008)
	5'-GAACCGACGACCCCCTGCTTGCAAAGCA-3'		
		
			

### Molecular assay for *Ehrlichia* spp.

Conventional PCR based on the *dsb* gene (400 bp) was used as the molecular detection assay for *Ehrlichia* spp. (Table 1). *Ehrlichia canis* (Jaboticabal strain GenBank access number by DQ401044) DNA was used as a positive control.

### Molecular assays for *Anaplasma* spp.

To detect DNA fragments from species of the genus *Anaplasma*, a nested PCR assay was performed, targeting approximately 932 bp of the 16S rRNA gene (Massung et al., 1998) (Table 1). DNA of bat blood (*Desmondus rotundus*) previously positive for *Anaplasma* spp. (Mello et al., 2024) was used as a positive control.

### Molecular assays for *Bartonella* spp.

A quantitative real-time PCR (qPCR) based on the nicotinamide adenine dinucleotide dehydrogenase subunit gamma (*nuoG*) gene was used as the molecular detection assay for *Bartonella* sp. The amount of *Bartonella* sp. DNA was quantified as *nuoG* copies per microliter, as proposed by André et al. (2016). The samples were analyzed in duplicate. When the difference of Cq (Cycle of quantification) values between the duplicates were higher than 0.5, the samples were retested in triplicate, following the MIQE guidelines (Bustin et al., 2009). To build the standard curve for each reaction, serial dilutions of different concentrations were made (ranging from 2.0 × 10^7^ to 2.0 × 10^0^ copies) of a plasmid that encodes an 83 bp fragment of the *B. henselae nuoG* gene (pIDTSMART; Integrated DNA Technologies, Coralville, Iowa, United States). The plasmid copy number was determined using the formula (XG/μL DNA/[Plasmid length (BP) × 660]) × 6.22 × 10^23^ × plasmid copies/μL. The amplification efficiency (E) was calculated using the formula E = 10^-1/slope^ of the standard curve, as described by Bustin et al. (2009). Negative controls for the reactions consisted of RNase-free distilled water (Thermo Scientific, Waltham, MA, USA). The qPCR assays were performed in a C1000-CFX96 thermal cycler (BIORAD, Headquarters: Hercules, CA, USA).

The qPCR-positive samples were subjected to additional PCR assays targeting *ribC* (420 bp) (Johnson et al., 2003), *gltA* (750 bp) (Norman et al., 1995), *rpoB* (800 bp) (Paziewska et al., 2011), and *groEL* (752 bp) genes (Zeaiter et al., 2002; Paziewska et al., 2011), as well as the 16S-23S rRNA ITS intergenic spacer region (453-717 bp) (Maggi et al., 2008) (Table 1). In the conventional PCR assays, ultrapure sterile water was used as a negative control and *Bartonella machadoae* DNA (Amaral et al., 2022a) as a positive control.

### Purification and sequencing

The PCR products were purified using a commercial kit (Wizard SV Gel and PCR Clean-Up System, Promega, Fitchburg, WI, USA), following the manufacturer’s instructions. The purified products were then stored in a freezer at -20 ºC until the moment they were subjected to sequencing. This procedure was carried out using the dideoxynucleotide chain termination method (Sanger et al., 1977) in an ABI PRISM 3700 DNA Sequence Analyzer (Applied Biosystems, Waltham, MA, USA) at the Center for Biological Resources and Genomic Biology – CREBIO (FCAV, UNESP Jaboticabal, SP, Brazil).

### Phylogenetic analysis

After sequencing, the electropherograms were analyzed using BioEdit v. 7.0.5.3 software (Hall, 1999) to examine the quality of the peaks corresponding to each nitrogenous base. A consensus sequence was built for each sample, and the BLAST online software program (Altschul et al., 1990) was used to compare the resulting samples with those deposited in the GenBank database (Sayers et al., 2025). The sequences were analyzed in FASTA mode and aligned with other homologous sequences of the same gene using the Clustal/W software via BioEdit v. 7.0.5.3 (Hall, 1999). The optimal nucleotide substitution model was determined using jModeltest v.2.1.10 software (Darriba et al., 2012). Bayesian inference was used as the phylogeny analysis method, employing MrBayes 3.2.2 software on XSEDE (Ronquist & Huelsenbeck, 2003) through the CIPRES portal (CIPRES, 2025).

## Results

### Search for inclusions by light microscopy

An analysis of the blood samples using light microscopy revealed no intraerythrocytic and intra-leucocytic inclusions that would suggest infection by hemoplasmas, *Bartonella* spp., *Ehrlichia* spp., and *Anaplasma* spp.

### Endogenous gene amplification

The 36 blood DNA samples showed satisfactory mammalian *gapdh* gene amplification.

### Hemoplasma detection and phylogenetic analysis

Five (16.7%) of the 36 samples tested positive for hemoplasmas by nested PCR, based on the 16S rRNA gene. Two (40%) of these five samples were positive by nested PCR for hemoplasmas based on the 23S rRNA gene (Table 2).

**Table 2:** Number of mammal specimens and species tested for *Mycoplasma* spp. by locality and host species, including positives/total per group.

**Family**	**Small mammal species**	**STM**	**MRB**	**MCP**	**TOTAL**
DIDELPHIDAE	*Didelphis marsupialis*	1/4 (16S rRNA)	2/6 (16S rRNA and 23S rRNA)	0/6	3/16
	*Didelphis imperfecta*	0/0	0/0	0/1	0/1
	*Philander opossum*	0/0	0/0	0/2	0/2
	*Marmosa murina*	0/0	0/0	0/7	0/7
	*Marmosa demerarae*	0/0	0/0	0/1	0/1
ECHIMYIDAE	*Mesomys hispidus*	0/0	0/0	0/4	0/4
	*Proechimys guyannensis*	0/0	0/0	1/1 (16S rRNA)	1/1
CRICETIDAE	*Nectomys rattus*	0/0	0/0	0/2	0/2
	*Oecomys bicolor*	0/0	0/0	1/1 (16S rRNA)	1/1
MURIDAE	*Mus musculus*	0/0	0/0	0/1	0/1
POSITIVE/TOTAL SAMPLES		**1/4**	**2/6**	**2/26**	**5/36**

STM: Santarém, PA; MRB: Marabá, PA; MCP: Macapá, AP.

The seven samples sent for sequencing resulted in only one readable 23S rRNA hemoplasma sequence (492 bp) from an individual of *Didelphis marsupialis* from Marabá, state of Pará.

The abovementioned sequence (GenBank access number OR943699) showed 99.36-100% identity (E-value = 0.0; Query cover = 92.0-95.0%) with hemotropic *Mycoplasma* spp. sequences obtained from *Didelphis albiventris* in southern Brazil (MW694786, MW694787, OP271903).

**Figure 3:**
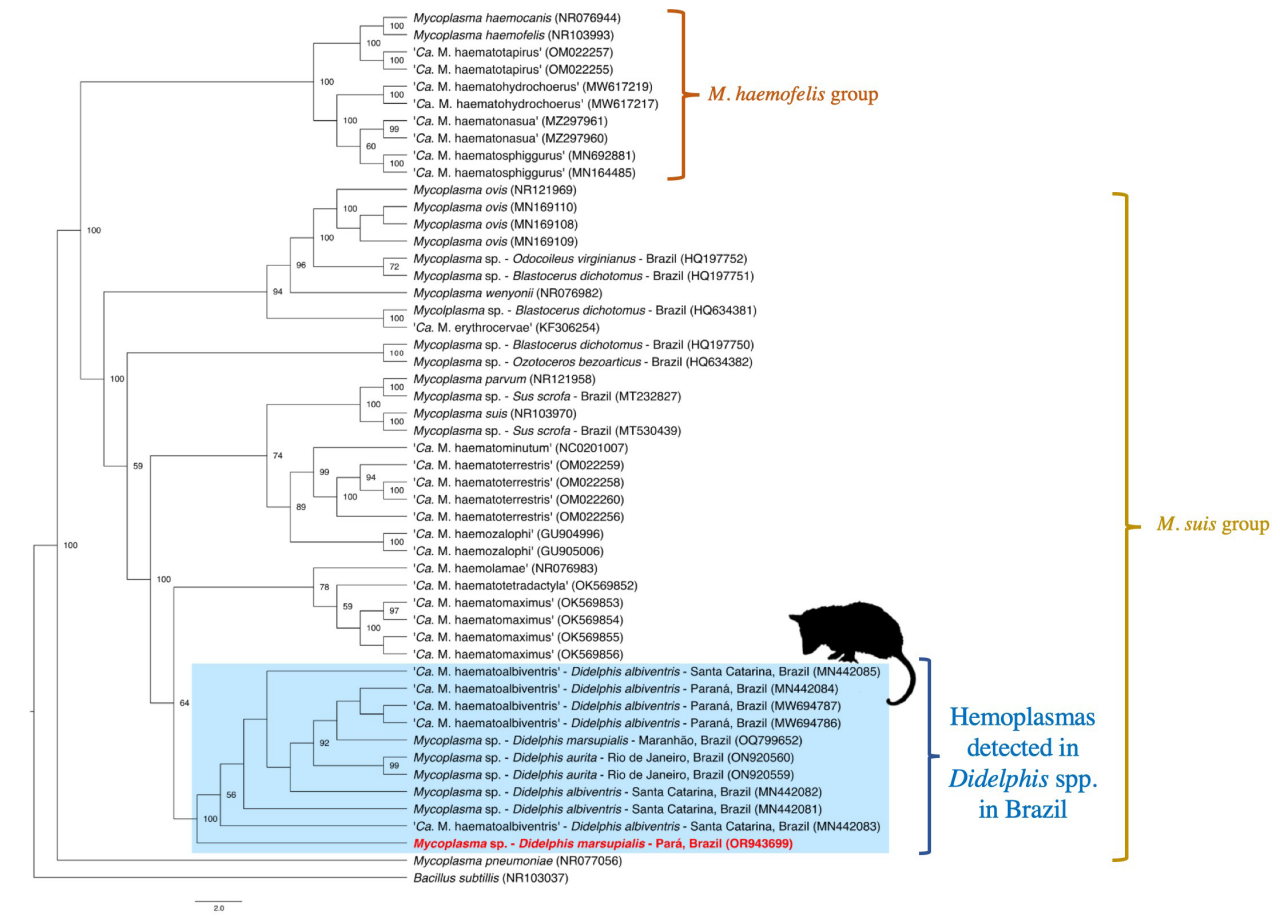
Bayesian phylogenetic tree based on an alignment of 540 bp of the 23S rRNA gene and GTR+G evolutionary model. A *Bacillus subtillis* sequence (NR103037) was used as an outgroup. The sequence obtained in this work is highlighted in red and is deposited in GenBank under Accession number. OR943699. Only posterior probability values of >50 are shown in the tree.

Using the Bayesian inference method, a phylogenetic tree was constructed based on the 23S rRNA gene (540 bp alignment) and GTR+G evolutionary model. The alignment included 49 homologous sequences deposited in GenBank, along with an outgroup (*Bacillus subtilis* – accession number. NR103037) and the sequence obtained in this study (Figure 3). The sequence obtained here formed a clade with other sequences previously detected in *Didelphis* spp. in Brazil’s south (MN442085, MN442083, MN442087, MN442086, MN442084, MN442081, MN442082), northeast (OQ799652), and southeast (ON920560, ON920559) regions.

### Molecular detection of *Ehrlichia* spp.

No sample showed amplification of the size expected for the protocol tested herein.

### Molecular detection of *Anaplasma* spp. and phylogenetic analysis

As expected, in the nPCR for *Anaplasma* spp., fragments of the 16S rRNA gene with compatible sizes were obtained from four small mammals, namely, two specimens of *D. marsupiali*s from Santarém and Marabá, PA, and one *D. masupialis* and one *Marmosa murina* from Macapá, AP (Table 3). The only readable sequence obtained from Marabá in *D. marsupialis* (GenBank number. PQ655482) showed 100% identity (E-value = 4e-162; Query cover = 99.69%) with *Anaplasma odocoilei* (GenBank access number. KT 870139.1) from white-tailed deer (WTD; *Odocoileus virginianus* in the United States).

**Table 3:** Number of mammal specimens and species tested for *Anaplasma* spp. by locality and host species, including positives/total per group.

**Family**	**Small mammal species**	**STM**	**MRB**	**MCP**	**TOTAL**
DIDELPHIDAE	*Didelphis marsupialis*	1/4	1/6	1/6	16
	*Didelphis imperfecta*	0	0	0/1	1
	*Philander opossum*	0	0	0/2	2
	*Marmosa murina*	0	0	1/7	7
	*Marmosa demerarae*	0	0	0/1	1
ECHIMYIDAE	*Mesomys hispidus*	0	0	0/4	4
	*Proechimys guyannensis*	0	0	0/1	1
CRICETIDAE	*Nectomys rattus*	0	0	0/1	2
	*Oecomys bicolor*	0	0	0/1	1
MURIDAE	*Mus musculus*	0	0	0/1	1
POSITIVE/TOTAL SAMPLE		**1/4**	**1/6**	**2/26**	**4/36**

STM: Santarém, PA; MRB: Marabá, PA; MCP: Macapá, PA; N/I: Number of individuals sampled/Number of individuals.

### Molecular detection of *Bartonella* spp. and phylogenetic analysis

Two individuals (5.5%) of two species, *D. marsupialis* (Didelphidae) and *Nectomys rattus* (Cricetidae), from the metropolitan region of Macapá, AP tested positive in the qPCR for *Bartonella* sp. (Table 4). The Efficiency, R^2^, Y-intercept and Slope values of the qPCR assays ranged from 94.1% to 98.5%, 0.999–0.993, 38.392–39.049 and − 3.437 to − 3.359, respectively. Quantification could not be estimated due to the difference of Cq higher than 0.5, even when running the samples in triplicate, possibly because of the Monte Carlo effect (Bustin et al., 2009). These two positive samples were negative in the additional PCR assays for *Bartonella* spp., likely due to the small amount of *Bartonella* DNA.

**Table 4:** Number of mammal specimens and species tested for *Bartonella* spp. by locality and host species, including positives/total per group.

**Family**	**Small mammal species**	**STM**	**MRB**	**MCP**	**TOTAL**
DIDELPHIDAE	*Didelphis marsupialis*	0/4	0/6	1/6 (qPCR)	16
	*Didelphis imperfecta*	0	0	0/1	1
	*Philander opossum*	0	0	0/2	2
	*Marmosa murina*	0	0	0/7	7
	*Marmosa demerarae*	0	0	0/1	1
ECHIMYIDAE	*Mesomys hispidus*	0	0	0/4	4
	*Proechimys guyannensis*	0	0	0/1	1
CRICETIDAE	*Nectomys rattus*	0	0	1/2 (qPCR)	2
	*Oecomys bicolor*	0	0	0/1	1
MURIDAE	*Mus musculus*	0	0	0/1	1
POSITIVE/TOTAL SAMPLE		**0/4**	**0/6**	**2/26**	**2/36**

STM: Santarém, PA; MRB: Marabá, PA; MCP: Macapá, PA; N/I: Number of individuals sampled/Number of individuals.

## Discussion

This study involved the first molecular detection of *Bartonella* spp., *Anaplasma* spp., and hemoplasmas in small non-volant mammals in green areas of large urban complexes in the Brazilian Amazon, specifically in the states of Pará and Amapá. Additionally, *Bartonella* spp. was detected for the first time in *D. marsupialis* and *N. rattus* in northern Brazil. Our study confirms the presence of *Bartonella* spp. in didelphids in the Neotropics, supporting the findings of Braga et al. (2023), who molecularly detected bartonellae in specimens of *D. marsupialis* in the state of Maranhão, northeastern Brazil.

According to Krügel et al. (2022), approximately 231 species of small non-flying mammals have been identified as reservoirs of *Bartonella* worldwide. However, only one study has detected *Bartonella* in blood samples from opossums in Brazil (Braga et al., 2023). Studies conducted in northeastern Brazil and central west Brazil were unsuccessful in detecting *Bartonella* spp. in the blood of didelphids (Fontalvo et al., 2017; Sousa et al., 2018; Gonçalves et al., 2020; Amaral et al., 2022b). Our study found similar results in the regions of Santarém and Marabá in the state of Pará. Recently, a combination of microbiological (pre-enrichment liquid culture followed by isolation in chocolate agar) and molecular (qPCR followed by conventional PCR assays and sequencing) approaches enabled the detection and description of *Bartonella harrusi* in wild marsupials (*Thylamys macrurus* and *Monodelphis domestica*) in the Pantanal of Mato Grosso do Sul (Amaral et al., 2022b). Future studies using these combined approaches are needed to achieve higher sensitivity in the methods used for *Bartonella* spp. detection in didelphids.

Species within the family Cricetidae, including those belonging to the genus *Nectomys*, have been identified as putative reservoirs of *Bartonella* spp. and have been found to harbor fleas with positive molecular detection for these bacteria in Brazil and Argentina (Gonçalves et al., 2016; Cicuttin et al., 2019; Schott et al., 2020). *Nectomys rattus* is widely distributed in the Neotropics, with confirmed occurrences in Brazil, Colombia, French Guiana, Guyana, Suriname, Venezuela, and Bolivia.Indeed, specimens of *N. rattus* were found to be positive for *Bartonella* spp. in the Cerrado biome in the states of Goiás and Mato Grosso do Sul (Gonçalves et al., 2016).

Although no molecular evidence of infection by *Ehrlichia* spp. was found in any of the ten host species tested in this study, these results do not completely rule out the possibility of infection in small non-flying mammals in the northern region of Brazil. Previously, varying positivity results for *Ehrlichia* spp. in small mammals were reported in Brazil’s Pantanal (Benevenute et al., 2017; Sousa et al., 2017) and Caatinga biomes. Based on phylogenetic inferences targeting the *gltA* gene and 23S-5S intergenic region, André et al. (2022) detected a novel *Ehrlichia* genotype in *D. albiventris* from the Brazilian Pantanal.

Domestic and wild animals are considered reservoirs and may favor the dissemination of *Anaplasma* species that cause diseases in animals and humans. Sampling of small non-flying synanthropic mammals is still scant in the Brazilian Amazon Forest, and studies carried out so far have not succeeded in detecting these agents (Benevenute et al., 2017; Braga et al., 2018, 2023). However, rodents have already been shown to be infected by *Anaplasma* spp. in the biomes of Pantanal (Sousa et al., 2017), Caatinga, Cerrado, and Atlantic Forest (Benevenute et al., 2017). In our study, we found unprecedented molecular evidence of infection by *Anaplasma* spp. in *Marmosa murina* and *D. marsupialis* in the states of Amapá and Pará, respectively, expanding the list of known free-ranging hosts in Brazil.

The BLASTn analysis of the DNA fragment found in our study shows high identity to *Anaplasma odocoilei*. This relationship of genotypic proximity to a deer-associated *Anaplasma* species was found earlier in small rodents in Brazil (Benevenute et al., 2017). In that study, *Anaplasma* 16S rRNA genotypes closely related to *A. odocoilei* were detected in *Sphiggurus villosus* (Atlantic Forest biome) and *Rattus rattus* (Caatinga biome). More recently, *Anaplasma* 16S rRNA genotype closely related to *A. odocoilei* was also detected in tapirs in the Cerrado and Pantanal biomes (Mongruel et al., 2024). On the other hand, phylogenetic reconstruction based on the 16S rRNA gene of *Anaplasma* sp. from wild and domestic mammals and associated ticks, analyzed in Brazil’s Pantanal biome, did not show proximity to *A. odocoilei* (Sousa et al., 2017). These findings may indicate a greater diversification of *Anaplasma* sp. genotypes across the various regions of Brazil, a fact that has not yet been well characterized due to the paucity of studies conducted in hard to reach areas, such as northern Brazil.

Although the PCR protocol used here to detect the 16S rRNA gene of hemoplasmas showed bands of the expected size in the small mammals *D. marsupialis*, *Proechimys* sp., and *Oecomys bicolor*, unfortunately no readable sequence was obtained Nonetheless, one readable 23S rRNA hemoplasma sequence was obtained from a specimen of *D. marsupialis*. Small mammals from the three regions under study, Santarém and Marabá (state of Pará) and Macapá (state of Amapá), tested PCR positive for hemoplasmas based on the 16S rRNA gene. Our study provides the second known record of hemotropic *Mycoplasma* spp. in *D. marsupialis*, and the first for northern Brazil. Although hemoplasmas have already been molecularly detected in *Oecomys* sp. in the Cerrado biome, in the state of Tocantins (Gonçalves et al., 2016), specimens of *Proechimys* sampled in the Amazon biome in the states of Pará and Mato Grosso proved to be negative in a previous study (Gonçalves et al., 2016).

Representatives of the genus *Didelphis* as hosts of hemoplasmas had already been recorded in earlier studies in Brazil, with the main focus on areas located in southern Brazil. *Didelphis albiventris* has been found to harbor hemotropic *Mycoplasma* spp. in several locations in the states of Paraná (Massini et al., 2019; Oliveira et al., 2022, Santa Catarina (Pontarolo et al., 2021), and Mato Grosso do Sul (Gonçalves et al., 2020. In addition, hemoplasmas were molecularly detected in *D. aurita* in the states of Minas Gerais (Orozco et al., 2022) and Rio de Janeiro (Oliveira et al., 2023a), in southeastern Brazil.

A phylogenetic analysis based on the 23S RNA gene placed the hemoplasma genotype detected here in the same clade as homologous sequences obtained from marsupials of the genus *Didelphis* in Brazil. The 23S rRNA gene has been used in several recent studies to characterize genetic fragments of hemoplasmas in wild animals in Brazil (Vieira et al., 2021; Oliveira et al., 2022; Braga et al., 2023). However, this study confirms that amplifying and sequencing the 23S rRNA gene can be a viable alternative to using the 16S rRNA gene when satisfactory results cannot be obtained with the latter.

Although the hemoplasma 23S rRNA sequence obtained herein was highly similar to sequences previously detected in *Didelphis* spp. in other regions of Brazil, further studies are needed to amplify additional molecular markers in order to unravel the diversity of hemoplasmas in didelphids in South America. Nevertheless, this study consolidates the uniqueness of the *Didelphis*-associated hemoplasma clade and contributes to the evolving construction of the phylogeny of hemoplasmas.

We believe that a broader sampling, outside of atypical environmental periods in the Amazon region, accessing a greater number of possible hosts, may reveal a higher prevalence of infection than that recorded in our study for hemotropic bacteria, combining the use of molecular and microbiological techniques (Amaral et al., 2022a, b). However, detection in synanthropic specimens may be affected by the frequent presence of domestic animals and the circulation of people and vehicles, favoring the permanence of more generalist groups adapted to urban environments such as didelphid opossums (Adler & Seamon, 1996; Jansen, 2002). Moreover, it should be noted that this study provides evidence of hemotropic bacteria circulating in the green areas of urban complexes in Brazil’s Amazon region.

The limitions of the present study should be acknowledged. Although the 16S and 23S rRNA genes of *Mycoplasma* spp., together with *Anaplasma* spp. 16S rRNA gene, were amplified in conventional PCR assays, most sequencing attempts produced poor-quality results, with chromatograms showing low resolution and overlapping peaks. Failure to obtain sequences of sactisfactory quality can be explained by different factors, such as low bacteremia, low DNA concentration after purification procedure, potential co-infection and nonspecific amplification, and partial degradation of the genetic material. These challenges are frequent in wildlife studies, particularly when working with small hosts, from which only limited volumes of blood can be obtained. The resulting short sequences may restrict the resolution of BLASTn matches and phylogenetic positioning, especially for *Anaplasma* spp. (Caudill & Brayton, 2022). Nevertheless, the detection of these hemoparasites remains relevant given the novelty of the synanthropic wild mammals sampled, and highlights the need for optimization of protocols protocol for wildlife research.

## Conclusions

Our study demonstrated the circulation of hemotropic *Mycoplasma* spp., *Anaplasma* spp., and *Bartonella* spp. in small non-flying mammals living in urban vegetation fragments in cities in Brazil’s northern Amazon region. Thus, we provide new information regarding the diversity of genotypes of hemopathogens circulating in urban complexes in the Amazon, underscoring the need for broader studies involving the detection and characterization of blood-borne bacteria associated with wild and synanthropic animals.

## Data Availability

All data are included in the article.
